# Telomere Interacting Proteins and TERRA Regulation

**DOI:** 10.3389/fgene.2022.872636

**Published:** 2022-04-08

**Authors:** Lara Pérez-Martínez, Tina Wagner, Brian Luke

**Affiliations:** ^1^ Institute of Molecular Biology (IMB), Mainz, Germany; ^2^ IMDEA Food Institute, Madrid, Spain; ^3^ Institute of Developmental Biology and Neurobiology (IDN), Johannes Gutenberg Universität, Mainz, Germany

**Keywords:** telomere, TERRA, senescence, yeast, R-loop

## Abstract

Telomere shortening rates inversely correlate with life expectancy and hence it is critical to understand how telomere shortening is regulated. Recently, the telomeric non-coding RNA, TERRA has been implicated in the regulation of replicative senescence. To better understand how TERRA is regulated we employed a proteomics approach to look for potential RNA regulators that associate with telomeric sequences. Based on the results, we have identified proteins that may regulate TERRA in both a positive and negative manner, depending on the state of the telomere. In this mini-review, we discuss and speculate about these data to expand our understanding of TERRA and telomere interactors with respect to telomere shortening dynamics.

## Introduction

Telomeres make up the terminal structures of linear chromosomes that protect chromosome ends and contribute to safe-guarding genome integrity (Arnoult and Karlseder, 2015). Unless maintained by telomerase, telomeres shorten upon each passage through S phase due to the end replication problem (Lingner et al., 1995). As a result, when telomeres reach a critically short length they activate a checkpoint-mediated cell cycle arrest, termed replicative senescence (Lundblad and Szostak, 1989; Bodnar et al., 1998; Campisi and Di Fagagna, 2007). In higher organisms, replicative senescence serves as a tumor suppressor that controls the number of divisions a cell can undergo (Campisi and Di Fagagna, 2007). The rate at which telomeres shorten must be tightly regulated, as the accumulation of senescent cells contributes to organismal aging (Baker et al., 2016; Hernandez-Segura et al., 2018). Uncontrolled telomere shortening may lead to the accumulation of senescent cells at a premature age. Indeed, when comparing across species, it appears to be the rate at which telomeres shorten, and not their absolute length, which correlates with lifespan (Whittemore et al., 2019). In budding yeast, one single critically short telomere is sufficient to trigger replicative senescence (Abdallah et al., 2009), whereas multiple short telomeres are needed in human cells (Kaul et al., 2012). Thus, in telomerase negative cells, it is imperative to repair the short telomeres that spontaneously arise in early population doublings to prevent accelerated senescence onset. In budding yeast, homology-directed repair (HDR) promotes telomere recombination at critically short telomeres and prevents premature senescence (Le et al., 1999; Fallet et al., 2014). HDR at telomeres is also important for the viability of post-senescence yeast cells, called ‘survivors’, and for cancer cells that lack active telomerase and rely on the Alternative Lengthening of Telomere mechanisms (ALT tumors) (Lundblad and Blackburn, 1993; Claussin and Chang, 2015; Sobinoff and Pickett, 2017). An understanding of HDR-mediated telomere maintenance is therefore critical to 1) provide mechanistic insights into senescence regulation and 2) to elucidate potential targets for cancers that rely on HDR for immortality.

Telomeres are transcribed by RNA polymerase II into Telomere Repeat-containing RNA (TERRA) (Azzalin et al., 2007; Luke et al., 2008; Schoeftner and Blasco, 2008; Feuerhahn et al., 2010). TERRA exists in both a “free” nucleoplasmic RNA form, but can also associate to telomeric chromatin as an RNA-DNA hybrid (R-loop) (Balk et al., 2013; Pfeiffer et al., 2013; Arora et al., 2014; Nanavaty et al., 2017). In wild type yeast, telomeric R-loops are transient at normal length telomeres and do not overtly affect telomere dynamics. However, at critically short telomeres TERRA and TERRA R-loops become stabilized and promote HDR-mediated repair, which in turn, plays a pivotal role in preventing the onset of premature senescence (Balk et al., 2013; Graf et al., 2017). TERRA R-loops are also important for the optimal growth rates of post-senescent survivors in yeast (Misino et al., 2018). In ALT cancer cells, TERRA R-loops promote repair at telomeres by inducing replication stress (Silva et al., 2021). For this reason, TERRA R-loop regulation becomes essential for optimal telomere maintenance in the absence of telomerase.

TERRA and R-loop levels inversely correlate with telomere length, suggestive of a regulatory mechanism to ensure low R-loop abundance at normal length telomeres and increased R-loop accumulation at short telomeres. However, even at short telomeres (where stable R-loops promote repair), the hybrids must also eventually be resolved, as hyper R-loop stabilization accelerates senescence rates in yeast (García-Rubio et al., 2018). In this regard, R-loop regulation at telomeres may be similar to their regulation at some double strand breaks (DSBs), where R-loops are both necessary for the initiation of DNA repair but even eventually need to be removed at a later stage to allow Rad51 loading (Ohle et al., 2016; D’Alessandro et al., 2018; Niehrs and Luke, 2020; Marnef and Legube, 2021). Together, these results suggest that telomere maintenance via HDR, may require a myriad of factors regulating R-loop levels (positively and negatively), recombination intermediates and telomere localization.

In order to identify novel telomere interactors we recently employed a proteomics-based approach to identify proteins that associate to telomeric sequences, in an RNA-dependent manner (Pérez-Martínez et al., 2020). Briefly, an oligonucleotide bait harboring telomeric sequences was incubated with cell extracts from both non-senescent and senescent yeast cells. This allowed us to identify a set of proteins that interact with telomeric-like sequences *in vitro* in the context of wild-type and senescent cells using mass spectrometry. Subsequently, we repeated the experiment in the presence of recombinant RNase A and RNase H, to identify the protein candidates that associate with telomeric-like sequences in an RNA-dependent manner in the context of senescent cells. This was important as TERRA may participate in the recruitment of proteins to telomeres in senescent cells, when TERRA levels are elevated (Graf et al., 2017). Furthermore, the identification of RNA-dependent telomere interactors may help to elucidate the telosome in other cellular contexts when TERRA levels are elevated, such as early S phase of the cell cycle (Graf et al., 2017). In the recent years, similar approaches have been used to identify telomere- and TERRA-interactors in human cells (Scheibe et al., 2013; Kappei et al., 2017; Bluhm et al., 2019; Viceconte et al., 2021). Here, we will discuss some of the identified telomere interactors and their potential implications in telomere/TERRA maintenance. Further characterization of their functions may contribute to our mechanistic understanding of telomere regulation during aging and cancer.

### RNA Binding Proteins and Helicases at Telomeres

Due to their established roles in R-loop dynamics, RNA binding proteins (RBPs) and helicases are interesting, and obvious, candidates for the regulation of TERRA and telomeric R-loops (Table 1) and make up the largest class of telomeric interacting proteins identified (Pérez-martínez et al., 2020). Similarly, a recent study showed that TERRA interactors in both human and mouse cells are largely made up of RNA binding proteins and helicases (Viceconte et al., 2021). RBPs are well-known genome stability factors, which bind nascent transcripts and prevent their re-hybridization to the template DNA. To this end, RBPs can prevent unscheduled R-loop formation and preserve genome integrity. However, it should be pointed out that RBPs can also bind and stabilize R-loops that have pre-accumulated at specific R-loop-prone loci (Gavaldá et al., 2016; García-Rubio et al., 2018; Pérez-martínez et al., 2020). Therefore, depending on the context, nascent transcription vs pre-stabilized R-loops, RBPs may either prevent the formation of R-loops or promote their stabilization, respectively. Similarly, a dual role in R-loop regulation has been suggested for certain helicases. Indeed, helicases such as human DDX1 and UPF1 have been implicated in both promoting R-loop formation at the IgG locus and at DSBs, respectively (Almeida et al., 2018; Ngo et al., 2021). Paradoxically, DDX1 has also been shown to remove R-loops at DSBs (Li et al., 2016) and UPF1 can remove TERRA from telomeres (Azzalin et al., 2007). Therefore, it is highly likely that, in addition to RNase H enzymes, helicases and RBPs also regulate R-loops at telomeres depending on telomere length. Some factors may contribute to ensuring low (transient) R-loop levels at wild-type length telomeres and others may promote R-loop accumulation (stability) at shortened telomeres.

**TABLE 1:** Highlighted yeast helicases and RBPs identified as telomere interactors.

		Function	Human Homolog	Telomere Association in Non-Senescent Cells	Telomere Association in Senescenct Cells	Protein Levels in Senescent vs. WT Cells
Helicases	Upf1	ATP-dependent RNA helicase	UPF1	+	+	decreased
	Dbp2	ATP-dependent RNA helicase of the DEAD-box protein family	DDX5, DDX17	+	+	decreased
	Dbp7	Putative ATP-dependent RNA helicase of the DEAD-box family	DDX31	+	+	decreased
	Dbp10	Putative ATP-dependent RNA helicase of the DEAD-box protein family	DDX54	+	+	decreased
	Sen1	ATP-dependent 5′ to 3′ RNA/DNA and DNA helicase	SETX	−	+	not changed
	Pif1	DNA helicase, potent G-quadruplex DNA binder/unwinder	PIF1	−	+	not changed
	Dbp1	ATP-dependent RNA helicase of the DEAD-box protein family	DDX1, DDX3X, DDX3Y, DDX4, DDX 41	−	+	not changed
	Dbp9	DEAD-box protein required for 27S rRNA processing; exhibits DNA, RNA and DNA/RNA helicase activities	DDX56	−	+	decreased
	Hcs1	DNA helicase associated with DNA polymerase alpha; stimulated by replication protein A	IGHMBP2	−	+	not changed
RBPs	Npl3	RNA-binding protein; promotes elongation, regulates termination, and carries poly(A) mRNA from nucleus to cytoplasm	hnRNPA1, SRSF factors	+	+	not changed
	Yra1	Nuclear polyadenylated RNA-binding protein; required for export of poly(A)+ mRNA from the nucleus	ALY1, POLDIP3	−	+	decreased

**FIGURE 1:**
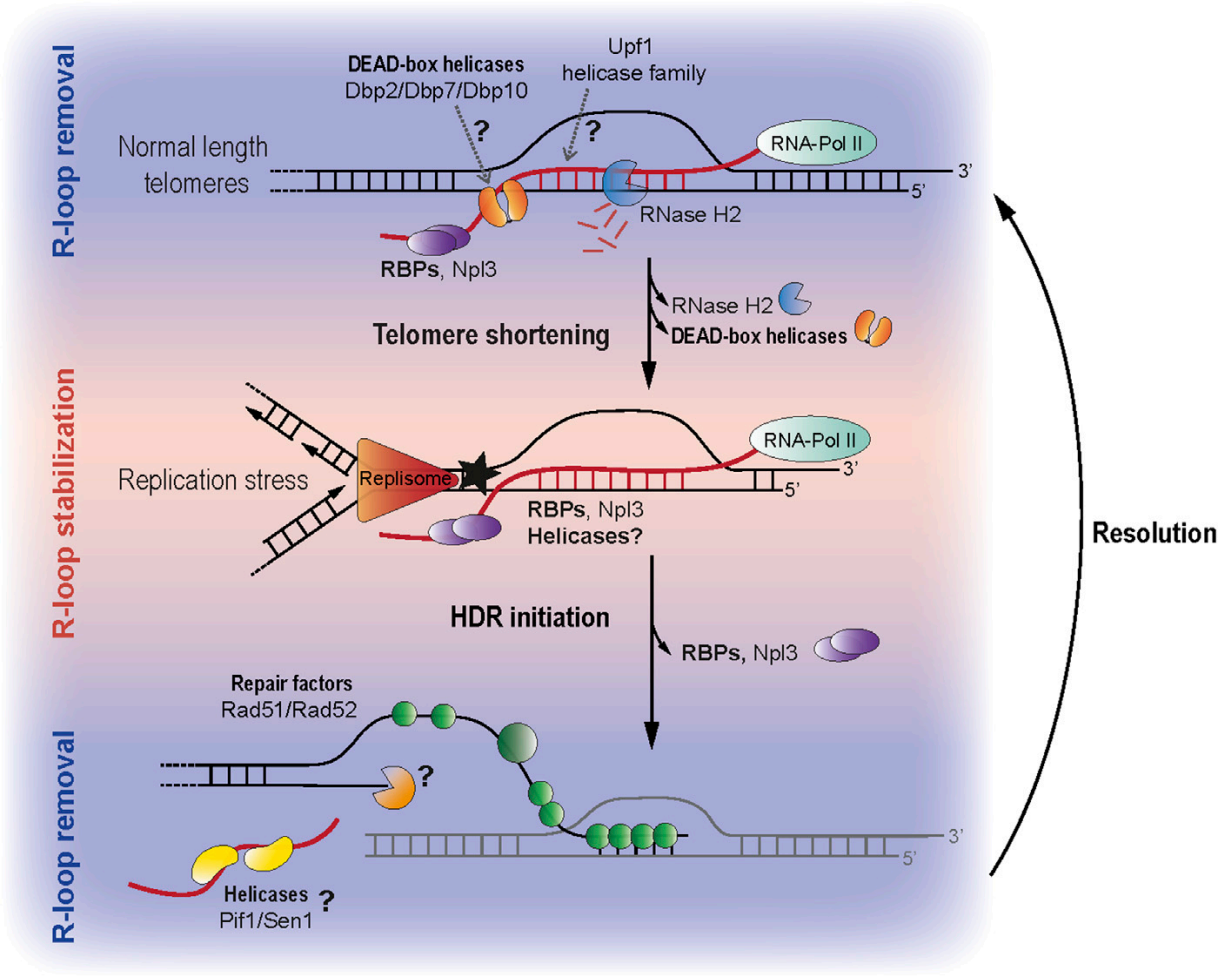
(Top) At normal length telomeres TERRA is transcribed and degraded in a cell cycle dependent manner. Along with RNase H2, other telomere interacting proteins (Dbps and Upf1 as well as the ssRNA binding protein Npl3) may contribute to the removal of TERRA (middle) When telomeres become critically shortened, TERRA R-loops accumulate at telomeres which promotes HDR mediated telomere maintenance. The absence of RNase H2 at short telomeres contributes to R-loop stability, however there may also be proteins which actively promote R-loop formation and stabilization. Npl3, for example can stabilize R-loops at short telomeres, how it changes from an R-loop preventer to promoter remains elusive. R-loops at short telomeres may drive replication stress and this may in turn trigger HDR. (bottom) Although telomeric R-loops are important to trigger HDR, it is likely that their removal is also required either to allow proper resection and/or re-annealing of the 3′ strand that was elongated. Helicases such as Pif1 and Sen1 are prime candidates to carry out such a function.

RBPs likely ensure balanced R-loop levels at telomeres through the binding of nascent TERRA transcripts, similar to their role at other transcribed loci (Niehrs and Luke, 2020). Helicases, on the other hand, may regulate TERRA R-loops by either removing, or promoting RNA:DNA hybrid structures. The regulation of TERRA and TERRA R-loops is most certainly dependent on the cellular context and telomere length status. In non-senescent budding yeast cells, TERRA hybrids remain transient to prevent replication stress and unscheduled HDR. Hence, RBPs and helicases found associated with telomeric sequences in non-senescent yeast extracts (Table 1; Figure 1 top) may represent a class of negative regulators of TERRA R-loops. This R-loop preventing function has been proposed in the past for several yeast RBPs (Pfeiffer et al., 2013; Santos-Pereira et al., 2013; Yu et al., 2014). In addition, mammalian hnRNPs (a class of RBPs) bind telomeres and TERRA to regulate telomere stability and length (LaBranche et al., 1998; Zhang et al., 2006; De Silanes et al., 2010; Redon et al., 2010; Flynn et al., 2011; Redon et al., 2013).

With regards to helicases, it is particularly interesting that we have identified yeast Upf1 as a telomeric interacting protein. In human cells it has been demonstrated that UPF1 facilitates telomere replication specifically on the leading strand (where TERRA hybridizes) in non-senescent cells (Chawla et al., 2011). Moreover, TERRA accumulates at human telomeres when UPF1 levels are reduced by shRNA (Azzalin et al., 2007). Together, it has been speculated that both human and yeast Upf1 may promote telomere replication through the removal of RNA-DNA hybrids. In yeast, the deletion of UPF1 results in very short telomeres (Askree et al., 2004; Gatbonton et al., 2006), however it remains to be determined if RNA-DNA hybrids play a role in this context. Recently, is has also been shown that human UPF1 can promote R-loop formation at subtelomeric DSBs, which in turn facilitates resection (Ngo et al., 2021). Together, these data highlight that the Upf1 protein is tightly intertwined with telomeres and RNA-DNA hybrid regulation, although the complete picture has not yet fully emerged. Indeed, it will be interesting to determine how Upf1 may be implicated in the regulation of TERRA R-loops.

Similar to Upf1, other identified yeast DEAD-box helicases, such as Dbp2, Dbp7 and Dbp10 might oppose telomeric R-loops in non-senescent yeast cells (Table 1). In particular, Dbp2, the yeast ortholog of DDX5, may limit R-loop accumulation at telomeres as it has been demonstrated to have RNA:DNA hybrid unwinding activity *in vitro* (Ma et al., 2013). Dbp2 regulates R-loop formation at the GAL locus (Cloutier et al., 2016) and associates to RNA transcripts within R-loop forming regions as well as to binding sites of the RNA/DNA helicase Sen1 (Senataxin in humans) (Tedeschi et al., 2018). Altogether, these data implicate Dbp2, as well as other yeast helicases associated with telomere sequences from non-senescent cells, in the negative regulation of telomeric R-loops. Interestingly, this regulation may be conserved in mammalian cells, as both human and mouse DEAD-box helicases (including DDX1 and DDX5) were shown to associate to TERRA-like sequences (Viceconte et al., 2021).

When telomeres shorten in telomerase-negative cells, TERRA and R-loops accumulate at telomeres (Churikov et al., 2016; Graf et al., 2017; Pinzaru et al., 2020). Interestingly, both RBPs and helicases may participate in these senescence-associated processes. At short telomeres, stable R-loops are important to promote HDR, recruit repair factors and prevent senescence onset (Balk et al., 2013; Graf et al., 2017; Pérez-martínez et al., 2020). Therefore, one could speculate that some of the helicases and RBPs associated to telomeric sequences from senescent extracts may help to promote or stabilize R-loops at telomeres (Table 1; Figure 1). However, if critically short telomeres behave in a manner to DSBs, then the hybrids would eventually have to be removed to facilitate Rad51 loading. Hence, it is feasible that helicases and RBPs associated with telomeric sequences from senescent extracts could either promote or oppose RNA-DNA hybrid formation.

Our recent data suggests that during replicative senescence Npl3 and other RBPs may participate in the R-loop stabilization process, perhaps by binding TERRA and reducing the accessibility of R-loop degrading enzymes to short telomeres (Figure 1) (Pérez-Martínez et al., 2020). Presumably, this may trigger replications stress and facilitate telomere elongation. Likewise, some of the identified yeast helicases binding to telomeres in the context of senescent cells may contribute to R-loop formation or stability, similar to human RTEL1 (Ghisays et al., 2021). Specific yeast helicases like Dbp1 or Dbp9 may either stabilize R-loops in the context of senescent cells or promote their formation in trans (Almeida et al., 2018). Indeed, Feretzaki et al. recently demonstrated that human TERRA can form telomeric R-loops in trans (Feretzaki et al., 2020). Therefore, it remains possible that some of the telomere binders identified in senescent cells participate in R-loop formation in trans. It is important to mention that, when using senescent yeast cell extracts, most of the telomere binding candidates identified associated to telomeric sequences in an RNA-dependent manner (Pérez-Martínez et al., 2020). This observation highlights the importance of RNA (presumably TERRA) in defining the telosome in senescent cells. Since mammalian TERRA sequences also associate with a myriad of helicases (Viceconte et al., 2021) and TERRA preferably forms R-loops at short telomeres (Graf et al., 2017; Feretzaki et al., 2020), it is likely that the TERRA-mediated recruitment of factors to short telomeres is conserved in higher organisms.

In order to allow R-loop accumulation and stabilization at shortened telomeres, R-loop-degrading factors would need to dissociate from telomeres while stabilizing factors are being recruited. This dissociation of R-loop stabilizers could either be a consequence of changes in their protein levels in senescent cells or due to lack of binding. Interestingly, the protein levels of the Dbp2, Dbp7 and Dbp10 helicases decrease in senescent yeast cells (Wagner et al., 2020), suggesting that their reduced abundance may allow R-loop accumulation (Figure 1). In this regard, these DEAD-box helicases could behave similar to RNase H2, which preferentially binds wild-type telomeres to degrade R-loops and decreases its interaction with short telomeres to allow R-loop accumulation (Graf et al., 2017). Altogether, the recruitment of potential R-loop stabilizers and the decreased binding of R-loop removers to short telomeres may combine to ensure R-loop stabilization. Similar to their role at DSBs, R-loops may act as scaffold structures to recruit repair factors to telomeres and promote HDR initiation (D’Alessandro et al., 2018) (Figure 1).

Once HDR has been initiated, R-loops need to be removed to successfully complete recombination and to allow Rad51 loading (Ohle et al., 2016; D’Alessandro et al., 2018; Marini et al., 2019; Niehrs and Luke, 2020; Marnef and Legube, 2021). To achieve this, both the dissociation of R-loop stabilizing factors as well as the recruitment of R-loop removing proteins may be required at telomeres (Figure 1). Indeed, over stabilization of telomeric R-loops negatively impacts senescence rates (García-Rubio et al., 2018). To counter-act this effect, R-loop stabilizers, such as Npl3, may dissociate from short telomeres via post-translational modifications by checkpoint kinases (Smolka et al., 2007). In addition, we suggest that, following HDR initiation, specific R-loop removing factors get recruited to critically short, recombining telomeres. In particular, the yeast helicases Sen1 or Pif1 are good candidates, as they associate with telomeric sequences specifically in the context of senescent cells (Pérez-Martínez et al., 2020) (Table 1).

Hence, once the recombination machinery is recruited to R-loops at short telomeres, the above-mentioned helicases may subsequently unwind R-loops to allow Rad51 loading (Figure 1 bottom) (Mischo et al., 2011; Pohl and Zakian, 2019; Schauer et al., 2020). In addition, helicases like Hcs1 may limit the formation of aberrant recombination intermediates, thereby coordinating telomere maintenance, similar to Srs2 in DSB repair (Putnam et al., 2010; Elango et al., 2017; Vasianovich et al., 2017). In summary, as a second step to RNA-DNA hybrid stabilization at short telomeres, other helicases may act to eventually remove RNA-DNA hybrids at critically short telomeres in an analogous manner to how helicases were described to clear hybrids for the efficient repair of DSBs (Li et al., 2016; Marnef and Legube, 2021).

It remains unclear how all of these factors are specifically recruited to short, recombining telomeres. Replication fork stalling at short R-loop harboring telomeres may recruit specific R-loop degrading factors like Pif1 (Schauer et al., 2020). Another possibility is that the displaced DNA strand of the R-loop recruits degrading factors, either through RPA binding (as in the case of RNase H1) or even recognition of the secondary structure of the ssDNA (Nguyen et al., 2017; Carrasco-Salas et al., 2019). Of note, the helicase activity of Hcs1 is stimulated by RPA (Biswas et al., 1993; Biswas E. E et al., 1997; Biswas S. B et al., 1997). Alternatively, it is possible that protein-protein interactions mediate the recruitment of R-loop degrading proteins. Certainly, some helicases like Dbp2 may interact with repair factors to ensure R-loop removal only after HDR has been initiated. In this respect, some helicases may behave similar to DDX5, which interacts with BRCA2 for DNA repair in human cells (Sessa et al., 2021).

In summary, there are likely a host of factors involved in the regulation of TERRA and its telomeric R-loops. Those that remove TERRA R-loops in non-senescent cells to prevent replication stress, those that ensure R-loop formation to identify critically short telomeres and induce HDR-initiating replication stress and finally, those that remove the hybrid to allow the progression of HDR (Rad51 loading).

### Other Factors at Telomeres

In addition to helicases and RBPs, other telomere binders identified may be implicated in HDR-mediated telomere maintenance in telomerase-null cells, among them the repair factor Mgm101 and the transcription factor Sdd4.

Mgm101, one of the strongest hits in the proteomics screen for telomere binders, plays a role in the maintenance of mitochondrial DNA but also participates in the nuclear DNA damage response (Chen et al., 1993; Rendeková et al., 2016). Interestingly, its repair function is linked to Mph1 (Ward et al., 2012; Rendeková et al., 2016; Silva et al., 2016), a known regulator of R-loops and replicative senescence (Lafuente-Barquero et al., 2017). In this context, Mph1 has been proposed to remodel replication forks at stable RNA-DNA hybrids. These findings, together with the slight upregulation of Mgm101 protein levels during senescence, raise the possibility that Mgm101 acts as an R-loop regulatory factor that balances senescence rate.

The transcription factor Sdd4 was identified as telomere-associated protein in WT cells (Pérez-Martínez et al., 2020). Its expression is induced in response to the DNA-damaging agent methyl methanesulfonate (MMS) (Lee et al., 2007; Yang et al., 2010). Sdd4 has recently been implicated in interchromosomal pairing of a specific locus in diploid cells (Kim et al., 2019). Having a predicted binding motif which is very similar to telomeric repeats (CCCCAC), one could speculate that Sdd4 also assists in the pairing of telomeres for their maintenance through HDR.

## Concluding Remarks

The identification and characterization of novel telomere factors remains essential to understand how telomere length is regulated. Since TERRA R-loops have been shown to regulate rates of telomere shortening (Balk et al., 2013) and senescence onset, it is also important to consider potential regulators of RNA-DNA hybrids. For this reason, the screening for telomere binders performed by Pérez-Martínez et al. is a powerful resource to study new players of telomere regulation. In particular, RBPs and helicases may be important factors for telomere length maintenance through TERRA R-loop regulation. Given the parallel mechanisms between short telomere maintenance and DSB repair, the identified telomere factors may also shed light onto DNA repair mechanisms.
